# Efficacy of switching from existing anti-vascular endothelial growth factor drugs to ranibizumab biosimilar in neovascular age-related macular degeneration

**DOI:** 10.1007/s10384-025-01224-0

**Published:** 2025-06-12

**Authors:** Hikaru Ota, Jun Takeuchi, Ryo Nonogaki, Kazuma Tamura, Hiroki Kaneko, Koji M. Nishiguchi

**Affiliations:** 1 Department of Ophthalmology, Nagoya University Graduate School of Medicine, 65 Tsurumai-cho, Showa-ku, Nagoya, Aichi 466-8550 Japan; 2 Department of Ophthalmology, Kyorin University School of Medicine, Tokyo, Japan; 3 Department of Ophthalmology, Hamamatsu University School of Medicine, Shizuoka, Japan

**Keywords:** Neovascular age-related macular degeneration, Ranibizumab biosimilar, Anti-vascular endothelial growth factor agent, Real-world outcomes

## Abstract

**Purpose:**

This study evaluated the clinical outcomes and aqueous humor cytokine levels in eyes with neovascular age-related macular degeneration (nAMD) switched from intravitreal aflibercept to ranibizumab biosimilar (BS).

**Study design:**

Prospective observational study.

**Methods:**

Thirty-eight eyes of 38 patients with nAMD who received aflibercept under a treat-and-extend (TAE) regimen were prospectively switched to ranibizumab BS. Eight eyes with cataracts undergoing surgery served as controls for aqueous humor cytokine analysis. Best-corrected visual acuity (BCVA) and anatomical outcomes were assessed over one year. The aqueous humor levels of vascular endothelial growth factor (VEGF)-A, angiopoietin-2 (Ang-2), and placental growth factor (PlGF) were measured before and after switching in eyes with nAMD and at surgery in controls.

**Results:**

Disease activity remained controlled in 94.3% of patients with nAMD for over one year. No significant changes were observed in the BCVA (*P*=0.65) after one year. Ang-2 levels remained unchanged (*P*=0.66) and were not significantly different between eyes with nAMD and controls both before (*P*=0.64) and after switching (*P*=0.30). PlGF levels also remained stable (*P*=0.12) but were significantly higher in eyes with nAMD than in controls both before (*P*<0.01) and after switching (*P*=0.03). VEGF-A levels significantly increased after switching (*P*<0.01) but remained lower than in the controls both before (P<0.01) and after switching (*P*=0.02).

**Conclusion:**

Switching from aflibercept to ranibizumab BS effectively maintained disease stability and cytokine balance in eyes with nAMD. These findings support ranibizumab BS as a viable and cost-effective alternative for long-term treatment.

## Introduction

Neovascular age-related macular degeneration (nAMD) is the leading cause of vision loss in older adults in developed countries [1, 2]. nAMD is a chronic inflammatory disease, and the interaction between various cytokines in the aqueous and vitreous humors plays a significant role in its pathogenesis. Key molecules involved in the development of nAMD include vascular endothelial growth factor (VEGF), placental growth factor (PlGF), and angiopoietin-2 (Ang-2). Elevated intraocular levels of these factors are reported to be closely associated with disease onset and responsiveness to therapeutic agents [3–5].

Intravitreal injection of anti-VEGF agents, including ranibizumab or aflibercept, considerably improves visual outcomes and reduces the incidence of blindness in patients with nAMD [6]. Additionally, treat-and-extend (TAE) regimens, which adjust treatment intervals based on the presence of macular exudation detected by optical coherence tomography (OCT), have been shown to effectively maintain long-term vision in patients with nAMD [7, 8].

However, a major concern regarding the long-term continuation of intravitreal anti-VEGF injections for nAMD is the substantial financial burden on both patients and healthcare systems due to the high cost of treatment. In the USA, the annual cost of anti-VEGF treatment for nAMD is estimated to be $10.7 billion [9].

Furthermore, multiple preclinical and clinical studies report that excessive suppression of intraocular VEGF below physiologically active levels may increase the risk of retinal pigment epithelium (RPE) dysfunction, retinal and choroidal blood flow impairment, and retinal atrophy[10–13].

A ranibizumab biosimilar (BS) developed by Senju Pharmaceutical Co., Ltd. (Senju Pharmaceuticals) was approved in September 2021 and subsequently introduced into the Japanese market. This product is the first biosimilar to an ophthalmic VEGF inhibitor in Japan. In Japan, the price of ranibizumab BS is approximately 70% that of the original ranibizumab, suggesting its potential to reduce the financial burden on both patients and healthcare systems.

In this study, we evaluated the clinical outcomes and aqueous humor cytokine levels in eyes with nAMD that were switched from continuous intravitreal aflibercept injections to ranibizumab BS to assess its efficacy and safety.

## Patients and methods

### Study design and approval

This prospective observational study was conducted in accordance with the tenets of the Declaration of Helsinki, approved by the Institutional Review Board of Nagoya University Graduate School of Medicine (2022-0104), and registered with the University Hospital Medical Information Network (UMIN000056994). Patients who met the inclusion criteria were informed of the study, and written informed consent was obtained prior to enrollment. All patient data were anonymized prior to analysis.

### Participants

The inclusion criteria were: patients with eyes diagnosed with nAMD, treated at Nagoya University Hospital between July 2022 and May 2024, in whom treatment was switched from aflibercept to ranibizumab BS. The decision to switch from aflibercept to ranibizumab BS was made by the physician when the patient was in a stable condition, defined as having been treated with aflibercept under a TAE regimen and maintaining a treatment interval of 16 weeks or longer for more than one year. Patients with age-related cataract were also included in the comparison of the aqueous humor cytokine levels. Patients with a history of diabetic retinopathy, cerebral or myocardial infarction, or vitrectomy were excluded.

All the patients underwent comprehensive ophthalmic examinations to support a multimodal imaging-based diagnosis. These examinations included best-corrected visual acuity (BCVA) measurements (logarithm of the minimum angle of resolution), fundus photography, fluorescein angiography, indocyanine green angiography (Spectralis HRA + OCT; Heidelberg Engineering, and spectral-domain optical coherence tomography (OCT) (Spectralis HRA + OCT; Heidelberg Engineering). Central retinal thickness (CRT) was manually measured on B-scan OCT images using a computer-based caliper tool integrated into an OCT system. CRT was defined as the distance from the inner limiting membrane to the Bruch’s membrane at the fovea on enhanced depth imaging OCT. Dry macula was defined as the absence of intraretinal, subretinal, and sub-RPE fluid, accompanied by either no hemorrhage or reduced hemorrhage.

### Procedure

**Fig. 1:**
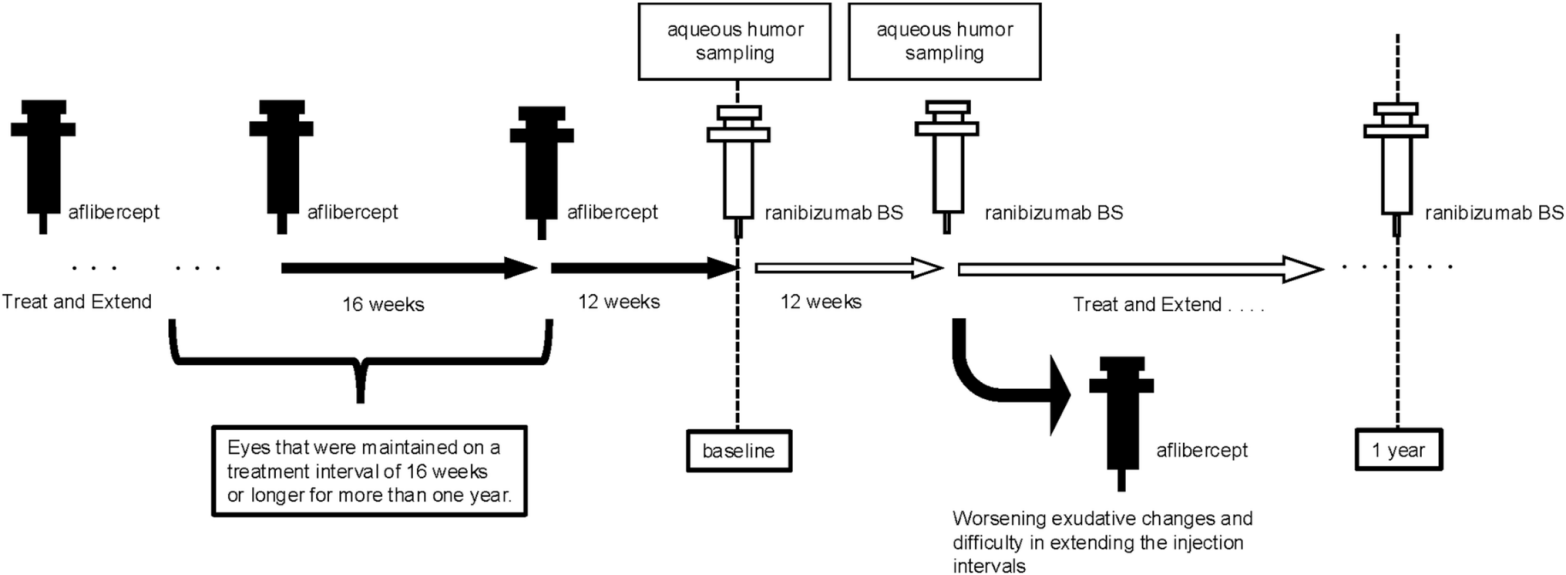
Switching protocol from aflibercept to ranibizumab biosimilar (BS) and timing of aqueous humor sampling in neovascular age-related macular degeneration (nAMD) cases

In the switching protocol for patients with nAMD, the treatment interval, which was ≧ 16

weeks, was shortened to 12 weeks during the transition from aflibercept to ranibizumab BS to minimize the risk of significant exacerbation of exudative changes following the switch. After switching, the injection interval was gradually extended with ranibizumab BS while monitoring exudative changes using OCT. If exudative changes worsened after switching and extending the injections’ interval in the TAE regimen with ranibizumab BS became difficult, a switch to aflibercept was performed based on the physician's decision. Clinical outcomes were assessed at predefined time points: immediately before and after switching from aflibercept to ranibizumab BS, and at 12 months post-switching. At the time of switching (under the influence of aflibercept) and after switching (under the influence of ranibizumab BS), approximately 0.10 mL of aqueous humor was collected via anterior chamber limbal paracentesis using a 27-gauge needle prior to the intravitreal injection of ranibizumab BS to evaluate the changes in intraocular cytokines associated with switching (under the influence of aflibercept) and after switching (under the influence of ranibizumab BS), approximately 0.10 mL of aqueous humor was collected via anterior chamber limbal paracentesis using a 27-gauge needle prior to the intravitreal injection of ranibizumab BS to evaluate the changes in intraocular cytokines associated with switching (Fig. 1). For patients with cataract, approximately 0.10 mL of aqueous humor was collected via 27-gauge limbal paracentesis prior to cataract surgery. Each aqueous humor sample was immediately transferred to sterile plastic tubes and stored at −80 °C until analysis.

### Measurement of cytokines using multiplex analysis

The concentrations of VEGF-A, PlGF, and Ang-2 in the aqueous humor were measured using the Luminex xMAP technology (Bio-Rad Laboratories Inc.) with the Milliplex® MAP Human Angiogenesis/Growth Factor Magnetic Bead Panel 1 assay (EMD Millipore). Aqueous humor samples (25 μL) were diluted two-fold and analyzed in accordance with the manufacturer’s protocol. Each sample was analyzed in a single run to ensure consistency. A standard control was included in the analysis, and the minimum detectable concentrations for VEGF-A, PlGF, and Ang-2 were 13.7 pg/mL, 1.4 pg/mL, and 13.7 pg/mL, respectively.

### Statistical analysis

All statistical analyses were performed using SPSS version 27 (IBM Corp.). The Shapiro–Wilk test was used to assess data normality. For group comparisons, Student’s t-test was used for normally distributed data, while the Mann–Whitney U test was used for non-normally distributed data. Comparisons between the baseline and each data point were performed using the Wilcoxon signed-rank test. The Bonferroni correction was applied to address multiple testing for comparisons between the baseline and each data point, in line with previous reports [14] . This involved calculating a corrected P-value threshold (0.05 divided by the number of tests), assuming the independence of each test. BCVA and CRT multiple time-point analysis results that fell below the Bonferroni correction threshold for multiple testing (P < 0.025, i.e., 0.05/2 tested exposures) were considered statistically significant. For the comparison of cytokine concentrations between the control and nAMD groups, Bonferroni correction was not performed, as this was a hypothesis-generating study aimed at identifying a possible association between AMD and cytokines, following the approach of previous reports on aqueous humor analysis [15] . Data are presented as the mean ± standard deviation, and for statistical tests other than the previously mentioned multiple comparisons P < 0.05 was considered significant.

## Results

### Demographic characteristics of the patients

A total of 38 eyes of 38 patients with nAMD were switched from aflibercept to ranibizumab BS during the study period. The baseline characteristics of the patients are summarized in Table 1, and the mean age was 76.5 ± 9.0 years. Treatment duration before switching to ranibizumab BS was 60.3 ± 25.3 months. At the time of switching, slight intraretinal fluid (IRF) overlying areas of retinal atrophy were observed in two eyes. Both eyes had been on a TAE regimen with aflibercept for over 16 weeks for more than one year. The IRF in these cases had been present for several years without notable progression during the extended treatment intervals. Based on previous reports, this IRF was considered as indication of atrophic cysts rather than a sign of macular neovascularization (MNV) activity, and the physician assessed these eyes as being in a stable condition [16–18] . Accordingly, switching to ranibizumab BS was performed (Table 2).

**Table 1:** Baseline characteristics of patients with nAMD switched to ranibizumab BS

38 eyes of 38 patients	
Age, years	76.5 ± 9.0
Male/female, eyes (%)	27 (71.1)/11 (28.9)
Subtypes of nAMD, eyes (%)	
Type 1 MNV	9 (23.7)
Type 2 MNV	6 (15.8)
PCV	22 (57.9)
Type 3 MNV	1 (2.6)
Treatment duration before switching to ranibizumab BS, months	60.3 ± 25.3
Treatment interval before switch to ranibizumab BS, weeks	16.7 ± 0.98

Data are presented as mean ± standard deviation

nAMD, neovascular age-related macular degeneration; MNV, macular neovascularization; PCV, polypoidal choroidal vasculopathy; BS, biosimilar

**Table 2:** Baseline characteristics of cases undergoing aqueous humor cytokine sampling

	nAMD	control	*P* value
Number of patients, eyes	30/30	8/8	
Age, years	76.5 ± 9.3	74.4 ± 9.0	0.57
Male/female, eyes (%)	22 (73.7)/8 (26.7)	3 (37.5)/5 (62.5)	0.09
PVD, eyes (%) Not complete/complete	6 (20.0)/24 (80.0)	1 (12.5)/7 (87.5)	0.54
Diabetes mellitus, eyes (%)	2 (6.67)	2 (25.0)	0.19
Subtypes of nAMD, eyes (%)			
Type 1 MNV	9 (30)		
Type 2 MNV	4 (13.3)		
PCV	17 (56.7)		
Type 3 MNV	0 (0)		

Data are presented as mean ± standard deviation

nAMD, neovascular age-related macular degeneration; PVD, posterior vitreous detachment; MNV, macular neovascularization; PCV, polypoidal choroidal vasculopathy

### Clinical outcomes after a single injection following the switch to ranibizumab BS

**Fig. 2:**
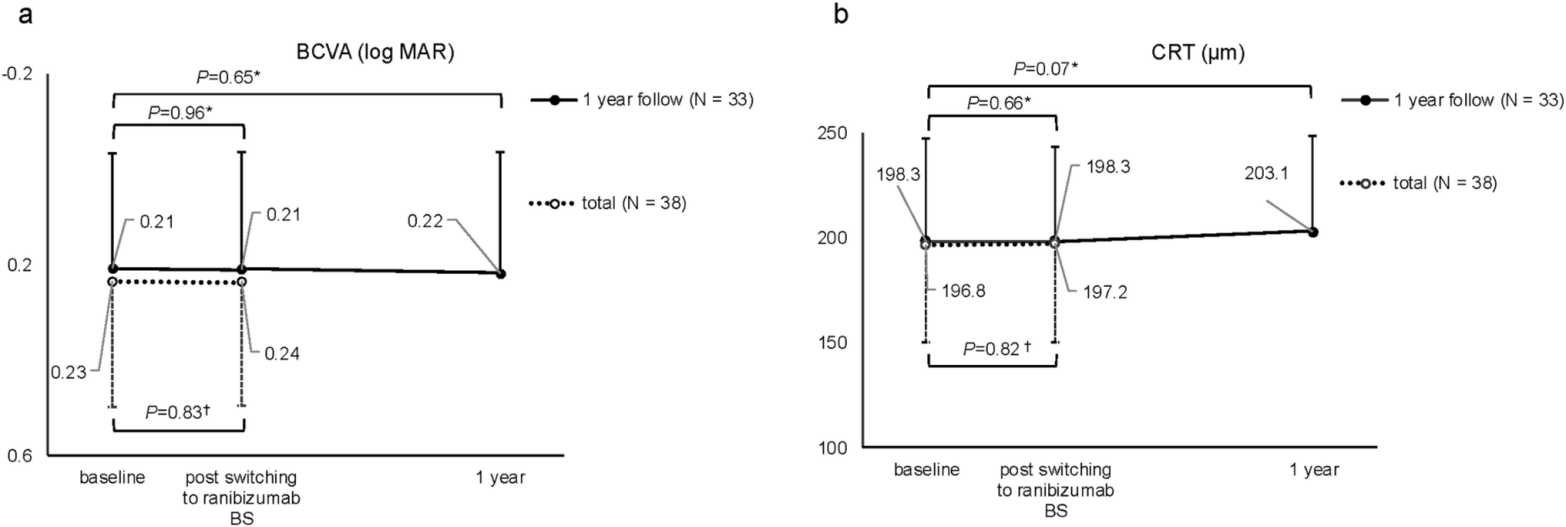
Changes in clinical outcomes before and after switching and one year post-switch. **a** Best-corrected visual acuity (BCVA); **b** central retinal thickness (CRT). The dotted line represents all 38 eyes that underwent switching, while the solid line represents 33 eyes that were followed up for more than one year. "Baseline" refers to 12 weeks after aflibercept administration, while "post switching to ranibizumab BS" refers to 12 weeks after ranibizumab biosimilar (BS) administration. *Wilcoxon signed-rank test and Bonferroni post-hoc test; ^†^Wilcoxon signed-rank test

All 38 eyes included in the study were available for comparison of clinical outcomes before and after switching from aflibercept to ranibizumab BS. The mean BCVA was 0.23 ± 0.26 at baseline and 0.24 ± 0.26 after switching, showing no significant difference (*P*=0.83). Similarly, the mean CRT was 196.8 ± 45.8 μm at baseline and 197.2 ± 46.8 μm after switching, with no significant changes observed (*P*=0.82) (Fig. 2). Regarding exudative changes, the proportion of patients achieving a dry macula was 36 of 38 eyes (94.7%) before switching and 34 of 38 eyes (89.5%) after switching.

### One-year clinical outcomes

During the one-year follow-up period, 3 eyes were lost to follow-up, leaving 35 eyes available for evaluation at the one-year mark. Among these, two eyes (5.71%) exhibited insufficient efficacy after switching to ranibizumab BS and were subsequently switched back to aflibercept. In one case, an 82-year-old man with type 3 macular MNV received four injections of ranibizumab BS but demonstrated persistent exudative changes at a 12-week injection interval, necessitating a switch back to aflibercept. In another case, an 80-year-old man with polypoidal choroidal vasculopathy (PCV) underwent two injections of ranibizumab BS but exhibited persistent exudative changes even at a 9-week injection interval, leading to a switch back to aflibercept. In the remaining 33 eyes that continued treatment with ranibizumab BS for one year, the mean BCVA was 0.21 ± 0.24 at baseline, 0.21 ± 0.25 after switching, and 0.22 ± 0.25 at one year, with no significant differences compared to baseline (*P*=0.96 and *P*=0.65, respectively). Similarly, the mean CRT was 198.3 ± 49.4 μm at baseline, 198.3 ± 45.0 μm after switching, and 203.1 ± 45.6 μm at one year, with no significant differences compared to baseline (*P*=0.66 and *P*=0.07, respectively) (Fig. 2). At baseline, the mean injection interval was 16.7 ± 0.98 weeks. It was initially shortened to 12 weeks for all cases per protocol but was extended to a mean of 16.0 ± 0.81 weeks one year after switching to ranibizumab BS. No cases showed worsening of visual acuity by more than 0.3 logMAR, nor were there any cases of subretinal hemorrhage leading to vision loss. Both eyes with IRF overlying areas of retinal atrophy at the time of switching maintained a stable condition over the one-year follow-up period. There were no significant changes in the atrophic areas or the IRF, and no apparent deterioration in visual acuity was observed.

### Analysis of aqueous humor cytokines

In the nAMD group, Ang-2 levels were 12.8 ± 29.1 pg/mL at baseline and 10.2 ± 16.6 pg/mL after switching, with no significant difference observed (*P*=0.66). In the control group, Ang-2 levels were 2.89 ± 4.53 pg/mL. No significant differences were observed between the control and nAMD groups at either baseline (*P*=0.64) or after switching (*P*=0.30).

PlGF levels in the nAMD group were 1.60 ± 1.71 pg/mL at baseline and 1.18 ± 1.50 pg/mL after switching, with no significant difference (*P*=0.12). In the control group, PlGF levels were 0.09 ± 0.14 pg/mL. Both baseline and post-switch PlGF concentrations in the nAMD group were significantly higher than those in the control group (*P*<0.01 and *P*=0.03, respectively).

**Fig. 3:**
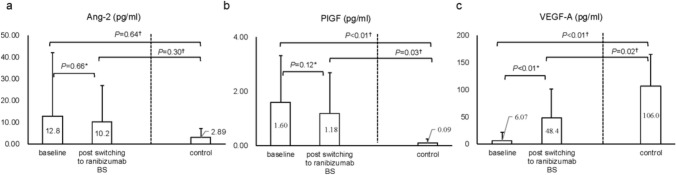
Aqueous humor cytokine levels in patients with neovascular age-related macular degeneration (nAMD) (33 eyes) and control subjects (8 eyes). **a** Angiopoietin-2 (Ang-2);** b** placental growth factor (PlGF); **c** Vascular endothelial growth factor-A (VEGF-A). "Baseline" refers to 12 weeks after aflibercept administration, while "post switching to ranibizumab BS" refers to 12 weeks after ranibizumab biosimilar (BS) administration. *Wilcoxon signed-rank test; ^†^Mann–Whitney U test

VEGF-A levels in the nAMD group were 6.07 ± 14.8 pg/mL at baseline and significantly increased to 48.4 ± 52.9 pg/mL after switching (*P*<0.01). In the control group, VEGF-A levels were 106.0 ± 59.1 pg/mL. Both baseline and post-switch VEGF-A concentrations in the nAMD group were significantly lower than in the control group (*P*<0.01 and *P*=0.02, respectively) (Fig. 3). There were no significant differences in aqueous humor cytokine levels at baseline, regardless of the history of diabetes or presence of posterior vitreous detachment (PVD).

## Discussion

This study shows that switching to ranibizumab BS maintained disease control for over one year in approximately 95% of patients with nAMD that had previously been stabilized with intravitreal aflibercept. Long-term administration of aflibercept using the TAE regimen is reported to effectively control exudative changes with an injection interval of 16 weeks or longer in 25.6%–47.0% of patients with nAMD [7, 8]. In such cases, treatment suspensions have been proposed, and recurrence rates of exudative changes are reported. According to Matsubara and Chujo, recurrence of exudative changes was observed in 50% of cases within two years after discontinuation of TAE and in 73.5% of cases within five years [19, 20]. Additionally, Arden et al. report that after discontinuation of TAE, exudative changes worsened in 29.4% of cases, with an average recurrence period of 14 months. Furthermore, a marked decline in visual acuity was observed upon recurrence compared to TAE discontinuation [21]. These reports indicate that TAE discontinuation is associated with a relatively high recurrence rate and that some patients experience visual deterioration upon recurrence, making continued treatment a frequently considered option.

However, long-term administration of high-cost anti-VEGF agents poses a substantial economic burden on both healthcare systems and patients. A study on patient preferences associated with anti-VEGF therapies found that, while visual prognosis was the top priority, treatment cost was the second most important factor. Therefore, physicians should consider the financial burden on patients when recommending the continuation of treatment [22, 23]. A study evaluating the cost-effectiveness of anti-VEGF therapy in Japan reports that if aflibercept was administered throughout a patient's lifetime to control disease activity following the onset of nAMD, the total cost would be JPY 2,075,183 (13,635 USD) higher than treatment with ranibizumab BS. In contrast, when aflibercept was used during the induction phase and ranibizumab BS during the maintenance phase, the cost increase was limited to JPY 291,822 (1,945 USD) [24]. The present study demonstrates that disease activity was successfully controlled in approximately 95% of cases over one year following the switch. Additionally, the mean treatment interval one year after switching was extended to 16.0 ± 0.81 weeks, indicating a remarkable benefit for the patient population included in the study.

In this study, we also evaluated the changes in the levels of aqueous humor cytokines following the switch in treatment. The concentration of Ang-2 showed no significant changes before and after switching. This result was pharmacologically reasonable because neither aflibercept nor ranibizumab BS had an inhibitory effect on Ang-2. Previous reports indicate that Ang-2 levels are higher in eyes with nAMD than in control eyes [4, 25]. In our study, Ang-2 levels tended to be elevated in eyes with nAMD both before and after switching compared to controls; however, the difference was not statistically significant.

Regarding PlGF, no significant changes were observed before and after switching; however, its levels remained considerably higher in eyes with nAMD than in control eyes at both time points. Previous studies report that PlGF levels are markedly elevated in eyes with nAMD compared to those in control eyes and suggest a potential association with the efficacy of anti-VEGF agents, consistent with the findings of this study [5, 26].

Previous studies report that aflibercept binds to and directly inhibits PlGF [27]. However, in the present study, no significant difference in the aqueous humor PlGF concentration was observed between aflibercept and ranibizumab BS administration adjusted at 12-week dosing intervals.

Although VEGF-A levels markedly increased following the switch from aflibercept to ranibizumab BS, the levels were still lower than in the controls. Aflibercept is reported to have a higher binding affinity for VEGF-A and a longer duration of suppression than ranibizumab; the changes in VEGF-A levels observed in this study are consistent with these findings [28–30]. Despite the observed increase in VEGF-A levels, the clinical course remained stable, with exudative changes successfully controlled for over one year in approximately 95% of the cases treated with ranibizumab BS. These results suggest that, while strong VEGF-A suppression is required during the induction phase of nAMD treatment, excessive VEGF-A suppression may not be necessary during the maintenance phase when disease activity is stable.

Previous studies using mouse models report that excessive suppression of VEGF-A leads to RPE atrophy, choriocapillaris occlusion, and cone cell dysfunction [10, 11]. Additionally, clinical studies, including randomized controlled trials and systematic reviews, demonstrate a correlation between the frequency of anti-VEGF injections and incidence of RPE atrophy [13, 31, 32]. Furthermore, a study by Cho et al. compared the efficacy of ranibizumab and aflibercept in Type 3 MNV and reports that the aflibercept group, which exhibited a stronger VEGF suppression effect than the ranibizumab group, had a higher incidence of retinal atrophy [33]. Based on these findings, it may be desirable to avoid excessive VEGF-A suppression and maintain appropriate intraocular VEGF-A levels to prevent exudative changes when managing the long-term disease activity of nAMD.

This study had some limitations. First, the sample size was relatively small, and the follow-up period was limited.

In particular, the number of control eyes undergoing cataract surgery for comparison of aqueous humor cytokine levels was eight, which may have resulted in weak statistical power.

Second, only one case of Type 3 MNV was included, making it challenging to compare treatment effects across different subtypes. Finally, this study included only patients with stable disease who were receiving aflibercept at intervals of 16 weeks or longer, suggesting a high level of safety associated with switching. However, caution is warranted when considering the application of this approach to eyes with high disease activity or insufficient VEGF suppression. Although no clinical deterioration in visual acuity or CRT was observed during the one-year follow-up period and the disease remained stable after switching, aqueous humor cytokine analysis revealed a significant increase in VEGF-A levels, indicating the need for continued long-term monitoring. On the other hand, a TAE discontinuation study involving patients with long-term stable exudative changes found that most recurrences occurred within the first year of a follow-up period ranging from two to five years [20, 21, 34]. In this context, the findings in the present study that 94.3% of eyes maintained exudative stability at one year may be considered a favorable outcome.

In summary, this study demonstrates that switching from aflibercept to ranibizumab BS in patients with nAMD receiving a TAE regimen effectively maintained disease control. Cytokine analysis of the aqueous humor revealed an increase in VEGF-A levels after switching; however, considerable suppression was maintained compared to normal eyes. These results suggest that in the maintenance phase of stable nAMD, switching to ranibizumab BS may be considered a cost-effective alternative.

## References

[1] Quartilho A, Simkiss P, Zekite A, Xing W, Wormald R, Bunce C. Leading causes of certifiable visual loss in England and Wales during the year ending 31 March 2013. Eye. 2016;30:602–7. 26821759 10.1038/eye.2015.288 PMC5108547

[2] Collaborators G 2019 B and VI, Adelson JD, Bourne RRA, Briant PS, Flaxman SR, Taylor HRB, et al. Causes of blindness and vision impairment in 2020 and trends over 30 years, and prevalence of avoidable blindness in relation to VISION 2020: the right to sight: an analysis for the Global Burden of Disease Study. Lancet Glob Health. 2021;9:e144–60.

[3] Bhutto IA, McLeod DS, Hasegawa T, Kim SY, Merges C, Tong P, et al. Pigment epithelium-derived factor (PEDF) and vascular endothelial growth factor (VEGF) in aged human choroid and eyes with age-related macular degeneration. Exp Eye Res. 2006;82:99–110. 16019000 10.1016/j.exer.2005.05.007 PMC4932847

[4] Ng DS, Yip YW, Bakthavatsalam M, Chen LJ, Ng TK, Lai TY, et al. Elevated angiopoietin 2 in aqueous of patients with neovascular age related macular degeneration correlates with disease severity at presentation. Sci Rep. 2017;7:45081. 28345626 10.1038/srep45081 PMC5366858

[5] Terao N, Koizumi H, Kojima K, Yamagishi T, Yamamoto Y, Yoshii K, et al. Distinct aqueous humour cytokine profiles of patients with pachychoroid neovasculopathy and neovascular age-related macular degeneration. Sci Rep. 2018;8:10520. 30002400 10.1038/s41598-018-28484-w PMC6043533

[6] Morizane Y, Morimoto N, Fujiwara A, Kawasaki R, Yamashita H, Ogura Y, et al. Incidence and causes of visual impairment in Japan: the first nation-wide complete enumeration survey of newly certified visually impaired individuals. Jpn J Ophthalmol. 2019;63:26–33. 30255397 10.1007/s10384-018-0623-4

[7] Ota H, Kataoka K, Asai K, Takeuchi J, Nakano Y, Nakamura K, et al. Five-year outcomes of treat and extend regimen using intravitreal aflibercept injection for treatment-naïve age-related macular degeneration. Graefes Arch Clin Exp Ophthalmol. 2024;262:3483–91. 38758378 10.1007/s00417-024-06519-5

[8] Ishibashi K, Haruta M, Ishibashi Y, Noda R, Dake S, Yoshida S. Four-year outcomes of intravitreal aflibercept treatment for neovascular age-related macular degeneration using a treat-and-extend regimen in Japanese patients. Ther Adv Ophthalmol. 2021. 10.1177/2515841420984586. 33506176 10.1177/2515841420984586 PMC7812394

[9] Siddiqui ZA, Dhumal T, Patel J, LeMasters T, Almony A, Kamal KM. Cost impact of different treatment regimens of brolucizumab in neovascular age-related macular degeneration: a budget impact analysis. J Manag Care Spec Pharm. 2022;28:1350–64. 36427338 10.18553/jmcp.2022.28.12.1350 PMC10373014

[10] Ford KM, Saint-Geniez M, Walshe T, Zahr A, D’Amore PA. Expression and role of VEGF in the adult retinal pigment epithelium. Investig Ophthalmol Vis Sci. 2011;52:9478–87. 22058334 10.1167/iovs.11-8353 PMC3250352

[11] Kurihara T, Westenskow PD, Bravo S, Aguilar E, Friedlander M. Targeted deletion of Vegfa in adult mice induces vision loss. J Clin Investig. 2012;122:4213–7. 23093773 10.1172/JCI65157 PMC3484459

[12] Nishinaka A, Inoue Y, Fuma S, Hida Y, Nakamura S, Shimazawa M, et al. Pathophysiological role of VEGF on retinal edema and nonperfused areas in mouse eyes with retinal vein occlusion. Investig Opthalmol Vis Sci. 2018;59:4701–13. 10.1167/iovs.18-23994 30267092

[13] Grunwald JE, Daniel E, Huang J, Ying G, Maguire MG, Toth CA, et al. Risk of geographic atrophy in the comparison of age-related macular degeneration treatments trials. Ophthalmology. 2014;121:150–61. 24084496 10.1016/j.ophtha.2013.08.015 PMC3892560

[14] Armstrong RA. When to use the Bonferroni correction. Ophthalmic Physiol Opt. 2014;34:502–8. 24697967 10.1111/opo.12131

[15] Sakamoto S, Takahashi H, Tan X, Inoue Y, Nomura Y, Arai Y, et al. Changes in multiple cytokine concentrations in the aqueous humour of neovascular age-related macular degeneration after 2 months of ranibizumab therapy. Br J Ophthalmol. 2018;102:448–54. 28765149 10.1136/bjophthalmol-2017-310284 PMC5890644

[16] Querques G, Coscas F, Forte R, Massamba N, Sterkers M, Souied EH. Cystoid macular degeneration in exudative age-related macular degeneration. Am J Ophthalmol. 2011;152:100-7.e2. 21570056 10.1016/j.ajo.2011.01.027

[17] Lai T-T, Hsieh Y-T, Yang C-M, Ho T-C, Yang C-H. Biomarkers of optical coherence tomography in evaluating the treatment outcomes of neovascular age-related macular degeneration: a real-world study. Sci Rep. 2019;9:529. 30679743 10.1038/s41598-018-36704-6 PMC6345958

[18] Zur D, Guymer R, Korobelnik J-F, Wu L, Viola F, Eter N, et al. Impact of residual retinal fluid on treatment outcomes in neovascular age-related macular degeneration. Br J Ophthalmol. 2025;109:307–15. 39033013 10.1136/bjo-2024-325640 PMC11866303

[19] Matsubara H, Matsui Y, Miyata R, Ichio A, Chujo S, Enomoto H, et al. Effects of suspension of anti-vascular endothelial growth factor treatment for neovascular age-related macular degeneration in clinical setting. Graefes Arch Clin Exp Ophthalmol. 2022;260:1867–76. 35094126 10.1007/s00417-021-05526-0 PMC9061688

[20] Chujo S, Matsubara H, Mase Y, Kato K, Kondo M. Recurrence rate during 5-year period after suspension of anti-vascular endothelial growth factor treatment for neovascular age-related macular degeneration. J Clin Med. 2024;13:4317. 39124583 10.3390/jcm13154317 PMC11312843

[21] Adrean SD, Chaili S, Grant S, Pirouz A. Recurrence rate of choroidal neovascularization in neovascular age-related macular degeneration managed with a treat–extend–stop protocol. Ophthalmol Retina. 2018;2:225–30. 31047590 10.1016/j.oret.2017.07.009

[22] Bhagat D, Kirby B, Bhatt H, Jager R, George M, Sheth V. Patient preferences associated with anti-vascular endothelial growth factor therapies for neovascular age-related macular degeneration and diabetic macular edema. Clin Ophthalmol. 2020;14:2975–82. 33061283 10.2147/OPTH.S273564 PMC7534869

[23] Ozdemir S, Finkelstein E, Lee JJ, Too IHK, Teo KYC, Tan ACS, et al. Understanding patient preferences in anti-VEGF treatment options for age-related macular degeneration. PLoS ONE. 2022;17: e0272301. 35951503 10.1371/journal.pone.0272301 PMC9371344

[24] Yanagi Y, Takahashi K, Iida T, Gomi F, Morii J, Kunikane E, et al. Cost-effectiveness analysis of ranibizumab biosimilar for neovascular age-related macular degeneration in Japan. Ophthalmol Ther. 2023;12:2005–21. 37171557 10.1007/s40123-023-00715-y PMC10287869

[25] Regula JT, von Leithner PL, Foxton R, Barathi VA, Cheung CMG, Tun SBB, et al. Targeting key angiogenic pathways with a bispecific CrossMAb optimized for neovascular eye diseases. Embo Mol Med. 2016;8:1265–88. 27742718 10.15252/emmm.201505889 PMC5090659

[26] Motohashi R, Noma H, Yasuda K, Kotake O, Goto H, Shimura M. Dynamics of soluble vascular endothelial growth factor receptors and their ligands in aqueous humor during ranibizumab for age-related macular degeneration. J Inflamm (Lond). 2018;15:26. 30534004 10.1186/s12950-018-0203-x PMC6280338

[27] Papadopoulos N, Martin J, Ruan Q, Rafique A, Rosconi MP, Shi E, et al. Binding and neutralization of vascular endothelial growth factor (VEGF) and related ligands by VEGF Trap, ranibizumab and bevacizumab. Angiogenesis. 2012;15:171–85. 22302382 10.1007/s10456-011-9249-6 PMC3338918

[28] Fauser S, Muether PS. Clinical correlation to differences in ranibizumab and aflibercept vascular endothelial growth factor suppression times. Br J Ophthalmol. 2016;100:1494–8. 26888975 10.1136/bjophthalmol-2015-308264

[29] Sawada T, Wang X, Sawada O, Saishin Y, Ohji M. Aqueous vascular endothelial growth factor and aflibercept concentrations after bimonthly intravitreal injections of aflibercept for age-related macular degeneration. Clin Exp Ophthalmol. 2018;46:46–53. 28621038 10.1111/ceo.13002

[30] Stewart MW. Intraocular drugs: pharmacokinetic strategies and the influence on efficacy and durability. Expert Opin Drug Metab Toxicol. 2024;20:977–87. 39258878 10.1080/17425255.2024.2401600

[31] Chakravarthy U, Harding SP, Rogers CA, Downes SM, Lotery AJ, Culliford LA, et al. Alternative treatments to inhibit VEGF in age-related choroidal neovascularisation: 2-year findings of the IVAN randomised controlled trial. Lancet. 2013;382:1258–67. 23870813 10.1016/S0140-6736(13)61501-9

[32] Eshtiaghi A, Issa M, Popovic MM, Muni RH, Kertes PJ. Geographic atrophy incidence and progression after intravitreal injections of anti-vascular endothelial growth factor agents for age-related macular degeneration. Retina. 2021;41:2424–35. 34101693 10.1097/IAE.0000000000003207

[33] Cho HJ, Hwang HJ, Kim HS, Han JI, Lee DW, Kim JW. Intravitreal aflibercept and ranibizumab injections for TYPE 3 neovascularization. Retina. 2018;38:2150–8. 28984737 10.1097/IAE.0000000000001862

[34] Aslanis S, Amrén U, Lindberg C, Epstein D. Recurrent neovascular age-related macular degeneration after discontinuation of vascular endothelial growth factor inhibitors managed in a treat-and-extend regimen. Ophthalmol Retina. 2022;6:15–20. 33775926 10.1016/j.oret.2021.03.010

